# Inhibition of mTOR complex 2 restrains tumor angiogenesis in multiple myeloma

**DOI:** 10.18632/oncotarget.25003

**Published:** 2018-04-17

**Authors:** Aurelia Lamanuzzi, Ilaria Saltarella, Vanessa Desantis, Maria Antonia Frassanito, Patrizia Leone, Vito Racanelli, Beatrice Nico, Domenico Ribatti, Paolo Ditonno, Marcella Prete, Antonio Giovanni Solimando, Francesco Dammacco, Angelo Vacca, Roberto Ria

**Affiliations:** ^1^ Department of Biomedical Sciences and Human Oncology, Internal Medicine Unit G. Baccelli, University of Bari Aldo Moro Medical School, Bari, Italy; ^2^ Department of Biomedical Sciences and Human Oncology, General Pathology Unit, University of Bari Aldo Moro Medical School, Bari, Italy; ^3^ Department of Basic Medical Sciences, Neurosciences, and Sensory Organs, Section of Human Anatomy and Histology, University of Bari Aldo Moro Medical School, Bari, Italy; ^4^ National Cancer Institute Giovanni Paolo II, Bari, Italy; ^5^ Hematology Unit, Di Venere Hospital, Bari, Italy

**Keywords:** angiogenesis, endothelial cells, multiple myeloma, mTOR, PP242

## Abstract

The mammalian Target of Rapamycin (mTOR) is an intracellular serine/threonine kinase that mediates intracellular metabolism, cell survival and actin rearrangement. mTOR is made of two independent complexes, mTORC1 and mTORC2, activated by the scaffold proteins RAPTOR and RICTOR, respectively. The activation of mTORC1 triggers protein synthesis and autophagy inhibition, while mTORC2 activation promotes progression, survival, actin reorganization, and drug resistance through AKT hyper-phosphorylation on Ser473. Due to the mTOR pivotal role in the survival of tumor cells, we evaluated its activation in endothelial cells (ECs) from 20 patients with monoclonal gammopathy of undetermined significance (MGUS) and 47 patients with multiple myeloma (MM), and its involvement in angiogenesis. MM-ECs showed a significantly higher expression of mTOR and RICTOR than MGUS-ECs. These data were supported by the higher activation of mTORC2 downstream effectors, suggesting a major role of mTORC2 in the angiogenic switch to MM. Specific inhibition of mTOR activity through siRNA targeting RICTOR and dual mTOR inhibitor PP242 reduced the MM-ECs angiogenic functions, including cell migration, chemotaxis, adhesion, invasion, *in vitro* angiogenesis on Matrigel^®^, and cytoskeleton reorganization. In addition, PP242 treatment showed anti-angiogenic effects *in vivo* in the Chick Chorioallantoic Membrane (CAM) and Matrigel^®^ plug assays. PP242 exhibited a synergistic effect with lenalidomide and bortezomib, suggesting that mTOR inhibition can enhance the anti-angiogenic effect of these drugs. Data to be shown indicate that mTORC2 is involved in MM angiogenesis, and suggest that the dual mTOR inhibitor PP242 may be useful for the anti-angiogenic management of MM patients.

## INTRODUCTION

Multiple myeloma (MM) is a hematologic malignancy characterized by the monoclonal expansion and accumulation of immunoglobulin-secreting plasma cells (PCs) within the bone marrow (BM) [1]. Angiogenesis plays an essential role in MM progression by ensuring tumor growth, PCs invasion and intramedullary/extramedullary dissemination [2]. In the BM milieu, a correlation is established between microvessel area and the rate of proliferating PCs: angiogenesis is enhanced in patients with active MM compared to those with non-active MM or with monoclonal gammopathy of undetermined significance (MGUS) [3], and MM PCs themselves trigger angiogenesis that in its turn sustains disease progression.

As emphasized by Hose *et al.* [4], at variance from memory B cells, normal BMPCs express a surplus of proangiogenic over antiangiogenic genes, resulting in induction of a basal level of *in vitro* angiogenesis. In MM patients, proangiogenic genes are aberrantly expressed and, conversely, antiangiogenic genes are down-regulated by MMCs, thus explaining the presence of BM angiogenesis to a variable extent in all of them. The accumulation of MMPCs would indeed gradually modify the BM microenvironment and sustain BM angiogenesis. Thus, targeting BM angiogenesis is an important therapeutic strategy. However, despite the development of new biologic agents such as bortezomib (the first-in-class proteasome inhibitor) and the immunomodulatory drugs (IMiDs), i.e. thalidomide and lenalidomide with established anti-angiogenic effects [5–7], MM still remains an incurable disease even if the time of relapse is remarkably delayed. Thus, the detection of new drugs, capable of simultaneously targeting tumor cells and angiogenesis, is eagerly awaited.

The Target of Rapamycin (TOR) is a serin-threonine protein kinase belonging to the phosphatidylinositol 3-kinase*-*related kinases (PIKKs) family [8]. In mammalian cells, TOR is named mammalian Target of Rapamycin (mTOR) that plays a central role in tumor growth, survival and drug resistance. The mTOR forms two different complexes: a) mTOR complex 1 (mTORC1 or C1), that is rapamycin-sensitive and consists of mTOR associated with RAPTOR (regulatory-associated protein of mTOR), mLST8, PRAS40 and Deptor; b) mTOR complex 2 (mTORC2 or C2), that is rapamycin-insensitive and binds RICTOR (rapamycin-insensitive companion of mTOR), mLST8, Sin1 and Protor. The two complexes play different roles in cellular metabolism: mTORC1 regulates protein translation, promoting cap-dependent mRNA translation through phosphorylation of S6K1 and 4EBP1 (eukaryotic translation initiation factor 4E binding protein-1) [9], and downregulates autophagy through its main target ULK1 [10]. mTORC2 activates serum glucocorticoid-regulated kinase (SGK) and protein kinase C (PKC) regulating actin cytoskeleton rearrangement, the activities of the small guanosine triphosphatases (GTPases) as RhoA, Cdc42 and Rac1 [11, 12], and phosphorylates its main target protein kinase B (AKT) on Ser473, that promotes cell survival, drug resistance and cell migration [13, 14]. Thus, the mTORC2 activity may represent a key process in promoting cancer cell invasion and metastases, as well as angiogenesis [14].

Preclinical data with first-generation mTOR inhibitors like rapamycin and rapalogs (such as temsirolimus, everolimus and deforolimus) showed their potential therapeutic activity in MM [15–17], but phase I/II clinical trials based on these drugs in combination with anti-MM drugs failed [18–20]. Furthermore, MM cell lines and primary MM PCs exhibit both mTORC1 and mTORC2 activation pathways [21]. Although there are currently no specific inhibitors of mTORC2, small molecules such as LY294002, PI-103, and NVP-BEZ235 are able to simultaneously target the adenosine triphosphate (ATP) binding site of both mTOR and PI3K [22, 23]. Accordingly, these inhibitors cannot be used to selectively inhibit mTOR and/or PI3Ks. Feldman *et al.* reported the synthesis of pyrazolo-pyrimidines (i.e., PP242 and PP30) that are the first dual, selective and ATP-competitive inhibitors of mTOR, with short *in vitro* half-maximal inhibitory concentrations (IC_50_ values) that are extremely selective for both mTOR complexes [24]. Moreover, PP242 was found to be effective on MM PCs by suppressing both mTORC1 and mTORC2 activities, thus resulting more effective as anti-cancer agent than rapamycin [25]. Additional data demonstrated higher mTORC2 activation in primary MM PCs and a synergistic effect of PP242 combined with bortezomib, strongly supporting the hypothesis that mTORC2 may be a new therapeutic target in MM [25].

Here, we demonstrate the activation of mTORC2 pathway in BM endothelial cells (ECs) from MM patients (MM-ECs) and its involvement in the regulation of MM-ECs angiogenesis. We have also addressed the dual mTOR inhibitor PP242 on MM-ECs to assess its anti-angiogenic activity in MM.

## RESULTS

### mTOR over-expression and hyper-activation in MM-ECs

**Figure 1:**
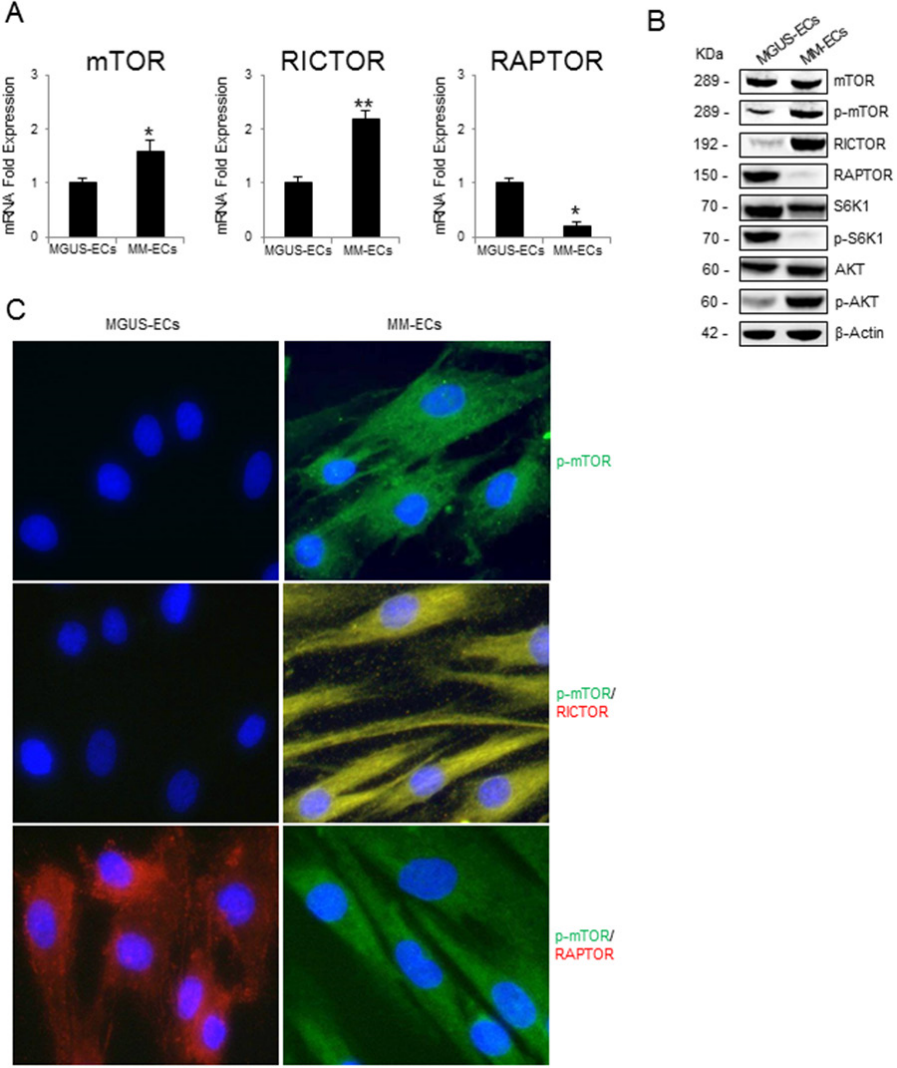
mTOR expression and mTOR activation pathway in MGUS-ECs and MM-ECs **(A)** Total mRNA was extracted from MGUS-ECs (n=12) and MM-ECs (n=20). mTOR, RICTOR and RAPTOR expression was evaluated by Real-Time RT-PCR and normalized to GAPDH. **(B)** Total proteins from MGUS-ECs (n=12) and MM-ECs (n=20) were analyzed by Western blotting to study mTOR, p-mTOR, RICTOR, RAPTOR, S6K1, p-S6K1, AKT, and p-AKT proteins expression in basal conditions. Immunoreactive bands were normalized to β-actin. Representative images of single MGUS-ECs and MM-ECs sample are shown. **(C)** MGUS-ECs and MM-ECs were cultured on chamber-slides, fixed and stained for p-mTOR (green), RICTOR (red) and RAPTOR (red). Nuclei were counterstained with 4’,6-diamidino-2-phenylindole (DAPI) (blue). Merge signal (yellow) shows co-localization. Original magnification 400X. Representative images from independent experiments carried out with MGUS-ECs (n=8) and MM-ECs (n=12) are shown. ^*^p < 0.03 and ^**^p < 0.003 by Wilcoxon signed-rank test.

To establish whether mTOR is more activated in MM-ECs than in MGUS-ECs, we examined mTOR mRNA and protein levels in both cells. As shown in Figure 1A, a significantly higher (+60%) mTOR mRNA expression was revealed by Real Time RT-PCR in MM-ECs than MGUS-ECs. When the expression of RAPTOR and RICTOR (that are essential for the activity of mTORC1 and mTORC2, respectively) was assessed, a significant increase of RICTOR was detected in MM-ECs (+120%), whereas RAPTOR resulted more expressed in MGUS-ECs (+80%).

When mTOR pathway was studied on Western blotting (Figure 1B), mTOR and RICTOR protein levels were found to be increased in MM-ECs but not in MGUS-ECs. On the contrary, RAPTOR protein was not found in MM-ECs. Moreover, an increased activation of mTOR was detected in MM-ECs in terms of phosphorylation at the Ser2448 [25]. Immunoblotting data highlighted an increase of total AKT (mTORC2 target) in MM-ECs, and a significant increase of its activation (p-Ser473). Instead, the main mTORC1 target S6K1 was more activated (p-Thr389) in MGUS-ECs than in MM-ECs. This was confirmed by immunofluorescence (Figure 1C): in MM-ECs, p-mTOR (green) co-localized with RICTOR (red), but not with RAPTOR (red).

These results indicate that mTORC2 pathway is more activated in MM-ECs than in MGUS-ECs and that it is likely involved in the regulation of MM-ECs activities.

### Effects of RICTOR knockdown in MM-ECs

To investigate whether mTORC2 is directly involved in MM angiogenesis, several functional assays using MM-ECs with RICTOR knocked-down were carried out. RICTOR silencing was performed through the siRNA transfection. By Real-Time RT-PCR and Western blotting, we assessed the optimal time and concentration of siRICTOR to ensure its inhibition. Supplementary Figure 1 shows that in MM-ECs treated with siRICTOR (25 nM for 72h) the RICTOR expression was strikingly reduced (-75% at both mRNA and protein levels). Immunoblotting analysis also revealed a significant reduction of both p-AKT and p-mTOR proteins in MM-ECs silenced for 72h (Supplementary Figure 1C).

**Figure 2:**
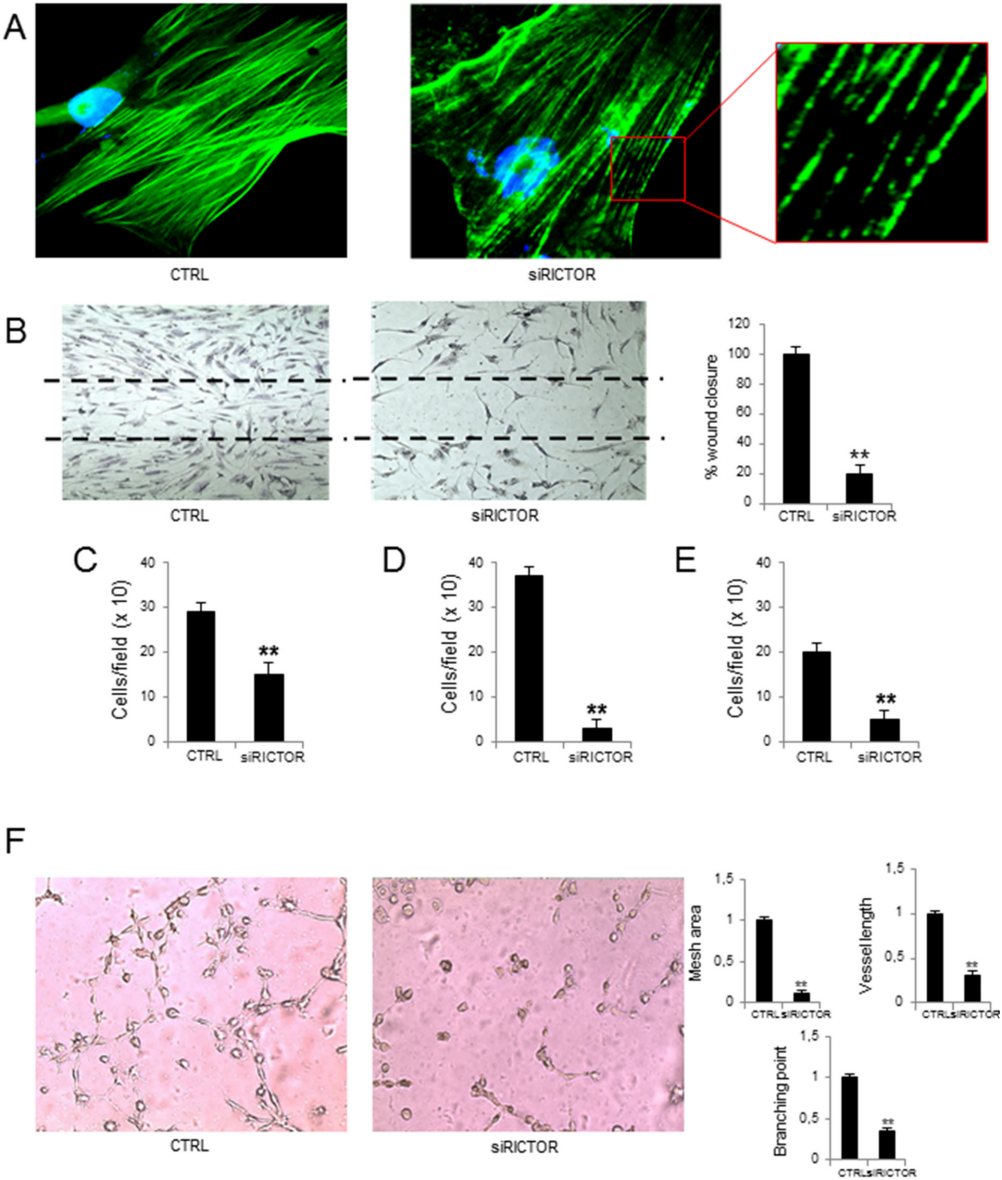
Effects of RICTOR knockdown on MM-ECs MM-ECs (n=8) were treated with scramble siRNA or with RICTOR siRNA (25 nM for 72h) and tested for **(A)** immunofluorescence to stain actin fibers with Phalloidin (green). Nuclei were counterstained with 4’,6-diamidino-2-phenylindole (DAPI) (blue). Original magnification 400X; **(B)** spontaneous migration to repair the scratch in the wound healing. Original magnification 200X. Data are expressed as mean±SD; **(C)** chemotaxis migration in response to chemoattractant agents (10 ng/mL FGF-2, 10 ng/mL VEGF and 1.5% FBS) in Boyden chamber. Data are expressed as mean±SD; **(D)** chemoinvasion in response to chemoattractant agents (10 ng/mL FGF-2, 10 ng/mL VEGF and 1.5% FBS) through Matrigel^®^ matrix. Data are expressed as mean±SD; **(E)** adhesion to fibronectin-coated 96-well plates of MM-ECs stained with Calcein AM. Data are expressed as mean±SD; **(F)** *in vitro* angiogenesis on Matrigel^®^ matrix-coated 48-well plates. Representative images from 8 independent experiments are shown. Original magnification 200X. Bar graphs indicate relative mesh area, vessel length and branching points, analysed by EVOS software. Data are expressed as mean±SD. ^**^p < 0.003 by Wilcoxon signed-rank test.

Figure 2A shows that, different from control cells whose intracellular Phalloidin-stained actin fibers appeared continuous and well-distributed, siRICTOR MM-ECs gave these fibers sparse and fragmented, indicating that RICTOR silencing interferes with actin reorganization. Also, compared to control cells that were able to fully repair the scratch, siRICTOR transfection reduced MM-ECs ability to repair a wound (-80%) (Figure 2B), to migrate in response to a chemical gradient of chemo-attractive agents, as shown by chemotaxis assay (-52%) (Figure 2C), to invade extra-cellular matrix (ECM; -92%) (Figure 2D) and to adhere to fibronectin (-75%) (Figure 2E).

Finally, control MM-ECs transfected with random siRNAs actively produced angiogenesis *in vitro*, based on a complex organization in which cells communicate to each other through the formation of a network with many junctions and branching points. On the contrary, MM-ECs transfected with siRICTOR were unable to form these networks. The capillaries on Matrigel^®^ were scanty and disorganized with few junctions, in the absence of empty area formation, as shown by the significant reduction of mesh area (-87%), branching points (-73%), and vessel length (-72%) (Figure 2F).

Experiments of RAPTOR knockdown were also performed, but results were not significant due to the low levels of RAPTOR in MM-ECs.

### Effects of PP242 treatment on MM-ECs

We next investigated whether the use of the dual mTOR inhibitor PP242 might have the same effects of RICTOR knockdown in MM-ECs. To find out the PP242 concentration with the highest inhibition of the functional abilities of MM-ECs, the wound-healing assay was performed with increasing concentrations of PP242. As shown in Supplementary Figure 2A, after treatment with PP242 at 10, 50, 100 and 200 nM, the mobility of MM-ECs was gradually reduced in step with the increased drug concentration. No effect on MM-ECs apoptosis and proliferation was observed (Supplementary Figure 2B and 2C). Western blot analysis of MM-ECs treated with PP242 (100 nM for 48h) showed a remarkable reduction of p-mTOR and p-AKT and a not significant effect on p-S6K1 and p-ERK1/2 (Supplementary Figure 2D), indicating that PP242 inhibits the mTOR pathway.

**Figure 3:**
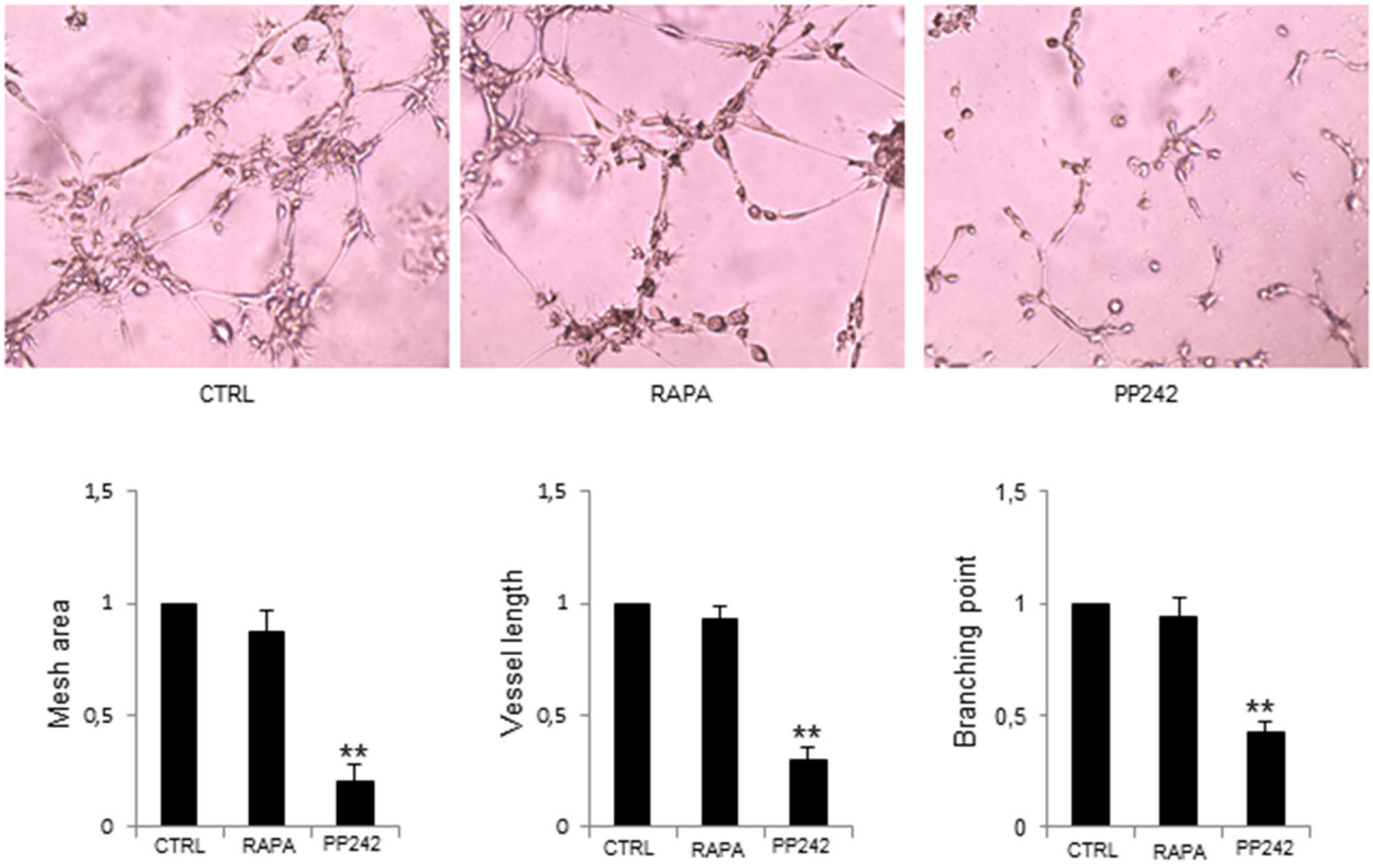
PP242 inhibition of MM-ECs angiogenesis *in vitro* MM-ECs (n=6) were treated with rapamycin (5 nM for 48h) or PP242 (100 nM for 48h) or untreated (CTRL) and tested for *in vitro* angiogenesis on Matrigel^®^ assay. Original magnification 200X. Representative images of 6 independent experiments are shown. Bar graphs indicate relative mesh area, vessel length and branching points analysed by EVOS software. Data are expressed as mean±SD. ^**^p < 0.003 by Wilcoxon signed-rank test.

Next, we evaluated the effect of PP242 on MM-ECs angiogenesis by Matrigel^®^ assay. Results were compared to those obtained with the mTORC1 inhibitor rapamycin (Figure 3). PP242 treatment reduced the constitutive MM-ECs capability to form capillary-like structures on Matrigel^®^ as shown by mesh area (-80%), vessel length (-70%) and branching points (-58%) inhibition, corroborating the role of mTORC2 in MM angiogenesis. By contrast, treatment of MM-ECs with rapamycin (5 nM for 48h) did not affect angiogenesis *in vitro* in line with the low expression of mTORC1 in MM-ECs.

**Figure 4:**
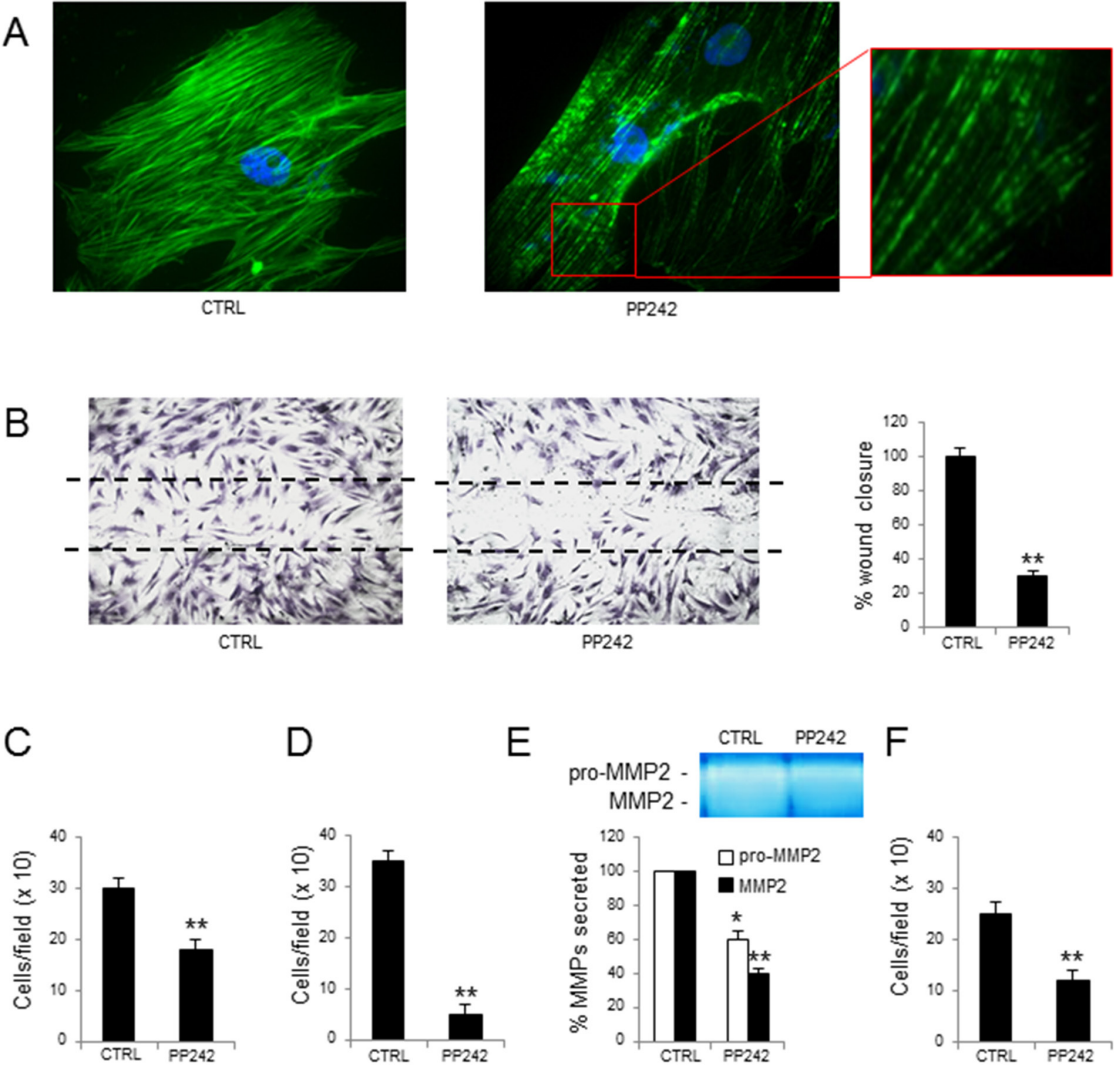
Effects of PP242 treatment on MM-ECs MM-ECs (n=10), treated or not with PP242 (100 nM for 48h), were tested for **(A)** immunofluorescence to stain actin fibers with Phalloidin (green), nuclei were counterstained with 4’,6-diamidino-2-phenylindole (DAPI) (blue). Original magnification 400X; **(B)** spontaneous migration to repair the scratch in the wound healing. Original magnification 200X. Data are expressed as mean±SD; **(C)** chemotaxis in response to chemoattractant agents (10 ng/mL FGF-2, 10 ng/mL VEGF and 1.5% FBS) in Boyden chamber. Data are expressed as mean±SD; **(D)** chemoinvasion in response to chemoattractant agents (10 ng/mL FGF-2, 10 ng/mL VEGF and 1.5% FBS) through Matrigel^®^ matrix. Data are expressed as mean±SD; **(E)** zymography of CM to determine the amount of active and inactive form of MMP2. Region of protease activity appeared as white bands and the percentage of MMPs release was estimated using Gel Logic 1,500 Imaging System. Data are expressed as mean±SD; **(F)** adhesion to fibronectin-coated 96-well plates of MM-ECs stained with Calcein AM. Data are expressed as mean±SD. ^*^p < 0.03 and ^**^p < 0.003 by Wilcoxon signed-rank test.

PP242 was able to alter actin organization, as revealed by immunofluorescence; at variance from controls, in MM-ECs treated with PP242 the F-actin appeared fragmented with gaps (Figure 4A). Moreover, PP242 reduced MM-ECs motility, as demonstrated by the reduction of spontaneous migration (-70%) (Figure 4B) and of chemotaxis ability (-40%) (Figure 4C). Given that the chemo-invasive property of treated MM-ECs was also decreased (-86%) (Figure 4D), we investigated whether the treatment with the PP242 was able to alter the secretion of metalloproteinases (MMPs) using the zymography assay. As shown in Figure 4E, the secretion of the inactive precursor of MMP-2 (pro-MMP-2) and the active form of MMP-2 were significantly reduced (-40% and -60%, respectively) in treated MM-ECs compared to controls. The adhesion assay also revealed a significantly reduced ability (-52%) of treated MM-ECs to adhere to fibronectin (Figure 4F).

### Effects of PP242 on angiogenesis *in vivo*

**Figure 5:**
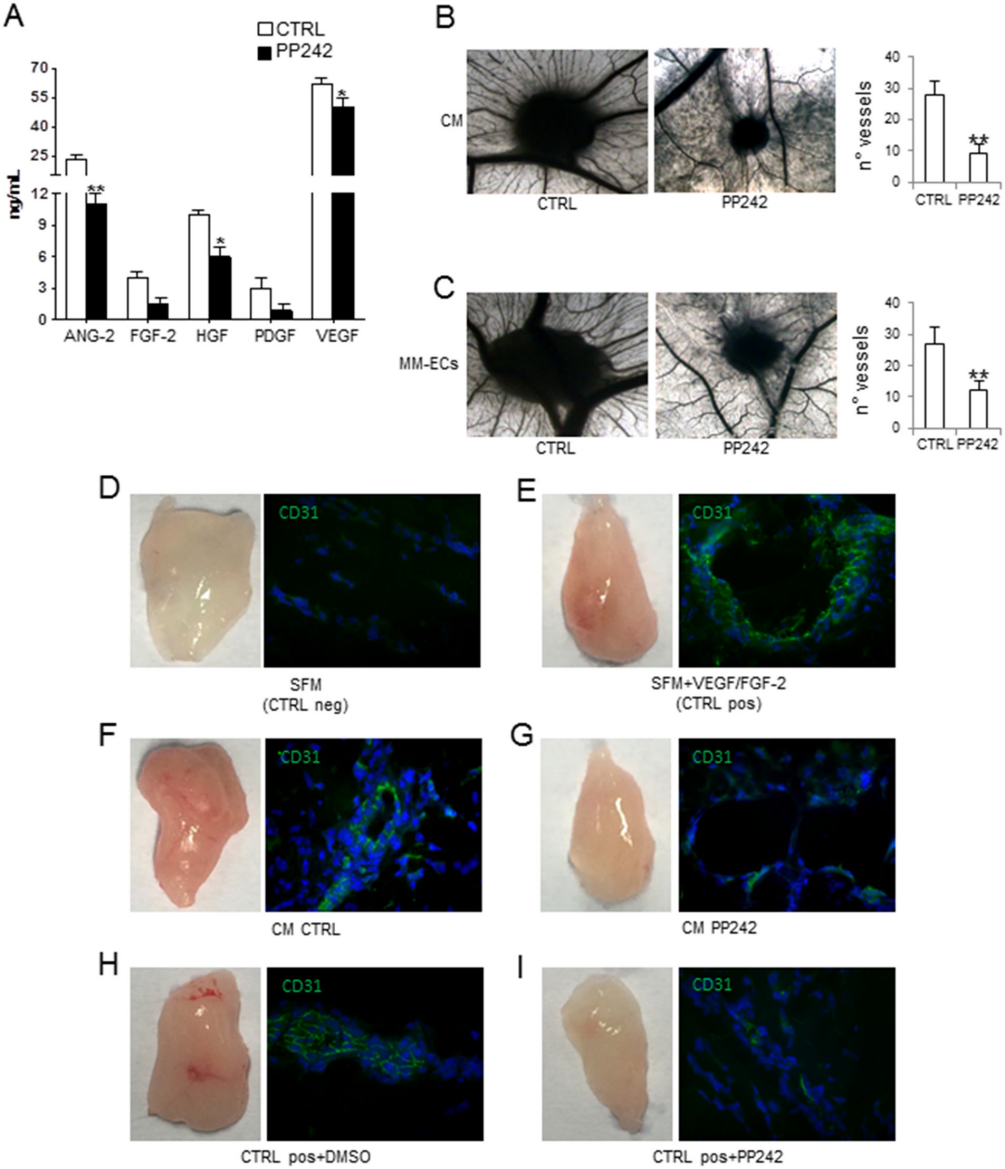
Effects of PP242 on angiogenesis *in vivo* Conditioned media (CM) from MM-ECs (n=16) treated or not with PP242 (100 nM for 48h) were tested for **(A)** pro-angiogenic cytokine release in a multiplex ELISA assay and **(B)** CAM assay. **(C)** CAM assay with MM-ECs (n=5) pre-treated or not with PP242 (100 nM for 48h). Original magnification 50X. Representative images from 5 independent experiments are shown. **(D-I)** Matrigel^®^ plug assay and immunofluorescence analysis of mouse CD31+ cells (green) from plug sections. NOD/SCID mice were injected with Matrigel^®^ containing: (D) serum free medium (SFM) as negative control; (E) SFM supplemented with VEGF/FGF-2 as positive control; (F) CM from untreated MM-ECs (n=4); (G) CM from MM-ECs (n=4) treated with PP242 (100 nM for 48h). (H-I) NOD/SCID mice injected with Matrigel^®^ containing SFM+VEGF/FGF-2 were intraperitoneally treated with (H) DMSO-vehicle or (I) PP242. Original magnification 200X. Representative images from 4 independent experiments are shown. ^*^p < 0.03 and ^**^p < 0.003 by Wilcoxon signed-rank test.

Preliminarily, we investigate the PP242 effects on the release of angiogenic cytokines by the Q-Plex assay. This specifically analyses the following pro-angiogenic cytokines: angiopoietin-2 (ANG-2), hepatocyte growth factor (HGF), interleukin-8 (IL-8), tumor necrosis factor-α (TNF-α), fibroblast growth factor-2 (FGF-2), platelet-derived growth factor (PDGF) and vascular endothelial growth factor (VEGF), and the anti-angiogenic molecules tissue inhibitor of metalloproteinase (TIMP) -1 and -2. Analysis of conditioned medium (CM) from MM-ECs, treated or not with PP242, showed a significant reduction of ANG-2, FGF-2, HGF, PDGF and VEGF released after treatment (Figure 5A).

We then asked the question whether PP242 was also able to inhibit *in vivo* angiogenesis. To this end, we performed the Chick chorioallantoic membrane (CAM) assay and the Matrigel^®^ plug assay. Figure 5B shows that CM from MM-ECs pre-treated with PP242 (100 nM for 48h) significantly inhibited *in vivo* angiogenesis: the number of vessels was 9±3 for CM from treated MM-ECs and 28±4 for untreated cells. Similar data were obtained testing CM from MM-ECs treated with PP242 for 24h that resulted in the formation of 11±2 vessels converging toward the sponge (data not shown). When we tested the direct effect of MM-ECs pre-treated with PP242 for 48h in the *in vivo* CAM assay (Figure 5C), we counted 27±5 vessels for control MM-ECs, while 12±3 for pre-treated MM-ECs. Finally, the anti-angiogenic effect of PP242 was analysed in NOD/SCID mice by Matrigel^®^ plug assay. Injection of CM from control MM-ECs attracted the mice CD31+ ECs into the plug and induced angiogenesis: plugs were macroscopically highly vascularized and appeared red colored (Figure 5F) in much the same way as in mice injected with Matrigel^®^ supplemented with FGF-2 and VEGF (positive control) (Figure 5E). Immunofluorescence analysis of CD31+ cells highlighted the organization of mice ECs into vessels (Figure 5E and 5F). CM from PP242-treated MM-ECs did not show the capability to attract CD31+ cells into plugs that were less colored (Figure 5G) in much the same way as in mice injected with Matrigel^®^ added with SFM (negative control) (Figure 5D). Furthermore, PP242 treatment of mice injected with Matrigel^®^ added with FGF-2 and VEGF (Figure 5I) showed that PP242 treatment inhibited migration of mice ECs and, thus, angiogenesis *in vivo* compared to vehicle-treated mice (Figure 5H).

### PP242 acts in synergy with anti-myeloma drugs

**Figure 6:**
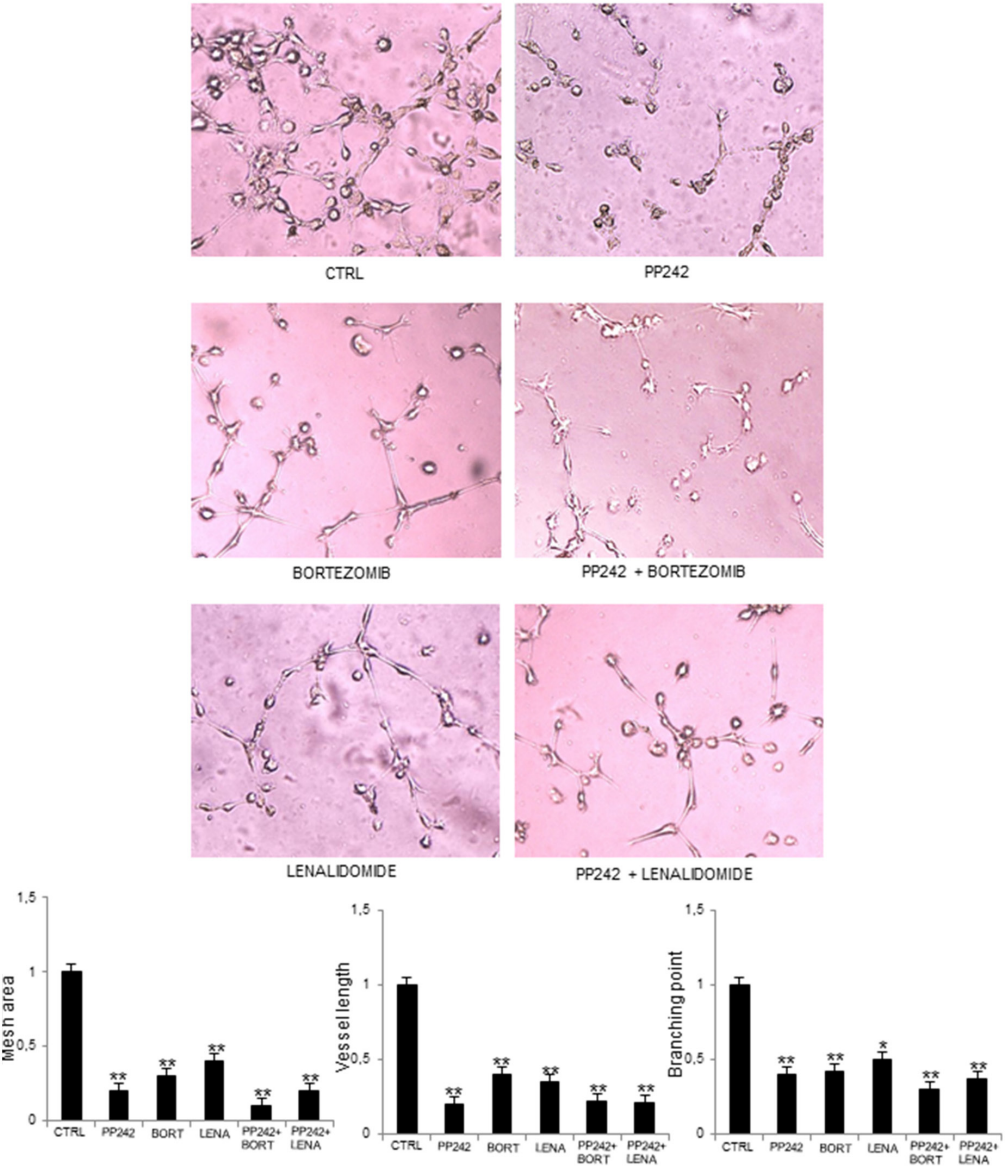
PP242 acts in synergy with other anti-myeloma drugs MM-ECs (n=10) were untreated or treated with PP242 (100 nM), bortezomib (7.5 nM) or lenalidomide (1.75 μM), alone or in combination, for 48h and tested for *in vitro* angiogenesis assay. Original magnification 200X. Bar graphs indicate relative mesh area, vessel length and branching points, analysed by EVOS software. Data are expressed as mean±SD. ^*^p < 0.03 and ^**^p < 0.003 by Wilcoxon signed-rank test.

Finally, we assessed the ability of PP242 inhibitor to act in synergy with two anti-myeloma and anti-angiogenic drugs, such as bortezomib [4] and lenalidomide [7]. The synergistic effects of PP242 with anti-MM drugs were tested by *in vitro* angiogenesis on Matrigel^®^ assay (Figure 6). PP242, bortezomib and lenalidomide alone were able to disrupt the network formation of capillaries on the Matrigel^®^ surface, compared to control MM-ECs. Interestingly, PP242 acted in synergy with anti-MM drugs: MM-ECs treated with PP242 in conjunction with bortezomib or lenalidomide showed a greater reduction of branches and capillary formation on Matrigel^®^ matrix compared to PP242, or bortezomib or lenalidomide alone, as demonstrated by the significant reduction of mesh area, vessel length and branching points.

## DISCUSSION

The role of mTOR pathway in tumor angiogenesis has been documented and associated to the activity of mTORC1 complex [26–28]. Indeed, the blockage of mTOR with rapamycin and rapalogs reduces tumor angiogenesis by inhibiting ECs functions and VEGF secretion [29]. Frost *et al.* demonstrated that the antitumor effect of rapamycin inhibitors *in vivo* is due to the down-regulation of VEGF secretion and the subsequent inhibition of angiogenesis [30].

Here we demonstrated that the activation of mTORC2 pathway is an important pro-angiogenic feature of MM-ECs. Our data highlighted an increase of mTOR phosphorylation on S2884, associated to a higher expression of RICTOR and to a rise in AKT phosphorylation on Ser473 in MM-ECs, indicating that mTORC2 is more activated than mTORC1 [12]. On the contrary, MGUS-ECs showed an increase of S6K1 phosphorylation as sign of mTORC1 activation, suggesting a shift from one complex to the other during the progression from MGUS to MM. RICTOR knockdown decreased MM-ECs cytoskeletal reorganization, migration, chemo-invasion, adhesion and *in vitro* angiogenesis on Matrigel^®^ assay, i.e., all cell functions needed for angiogenesis to develop in MM [31, 32].

In spite of mTOR crucial role in tumor progression, the first generation of mTOR inhibitors (rapamycin and rapalogs) have shown limited effects in phase I/II clinical trials in solid tumors [33–36]. In MM, clinical trials based on rapalogs in combination with anti-myeloma drugs have shown low activity [17–19], plausibly due to a negative feedback loop between mTORC1 and mTORC2 [13]. Inhibition of mTORC1 by rapamycin results in the hyperactivation of p-AKT pathway, by the stimulation of mTORC2 and, consequently in enhanced survival and chemoresistance of MM-PCs [13, 20, 37]. Based on these observations, mTOR inhibitors blocking both mTORC1/2 complexes have been developed. Promising preclinical studies showed a greater anti-proliferative and pro-apoptotic effect of the dual mTORC1/2 inhibitors compared to rapamycin. Some of these compounds, i.e. INK-128, MLN0128, TAK-228 are in phase I/II of clinical trials in advanced solid tumors, lymphomas as well as in MM [38, 39].

Given that MM-ECs show activated mTORC2 signaling and are insensitive to rapamycin, we wondered whether PP242 is a possible anti-angiogenic drug in MM. PP242 is a pyrazolo-pyrimidine belonging to the class of dual mTOR inhibitors that are able to block specifically mTORC1 and mTORC2 [24]. PP242 treatment at non-cytotoxic concentration inhibited angiogenesis both *in vitro* and *in vivo*. Accordingly, to the regulation of Rho and cytoskeleton reorganization mediated by mTORC2 [40, 41], PP242 alters actin reorganization that affects migration, chemotaxis, chemoinvasion and adhesion of MM-ECs. Reduction of cell adhesion on fibronectin was not due to a down-regulation in the expression of the α_v_β_3_ (data not shown), which is the most expressed integrin by MM-ECs [42, 43]. Perhaps the alteration of F-actin fibers, that interact with the C-terminal region of β-integrins favoring their integrity and functions [44], affects the interplay with α_v_β_3_ integrin and, as a consequence, its activity.

Activation of the PI3K/AKT/mTOR pathway regulates the release of VEGF and of others pro-angiogenic factors, i.e. nitric oxide, angiopoietins [45] and of MMP-2 and MMP-9 [46]. Accordingly, the PP242 treatment significantly reduced the secretion of ANG-2, VEGF, FGF-2, HGF and of pro-MMP-2 and active MMP-2. Both VEGF and HGF are implicated in MM angiogenesis due to the existence of autocrine and paracrine loops in MM that further activate MM-ECs [47, 48]. ANG-2 promotes ECs proliferation, migration, sprouting, and neovascularization in response to VEGF [49–51], and its inhibition reduces tumor angiogenesis. In addition, the inhibition of MMP-2 release along with the interference on cytoskeleton rearrangement contributes to the decreased MM-ECs chemoinvasion.

According to the reduction of release of pro-angiogenic cytokines, the CM from PP242-treated MM-ECs was unable to induce angiogenesis in the *in vivo* CAM and Matrigel^®^ plug assays. Conversely, CM from untreated MM-ECs induced vessel formation in both *in vivo* assays. Furthermore, the anti-angiogenic effect of the drug on MM-ECs angiogenic activities, i.e. migration, chemotaxis and angiogenesis, was confirmed *in vivo* by intraperitoneally treated NOD/SCID mice with PP242. Taken together, the *in vitro* and *in vivo* results highlight the importance of mTORC2 in the regulation of MM angiogenesis suggesting the PP242 as a new possible anti-angiogenic molecule in MM.

In line with our study, by using RAPTOR and/or RICTOR knockdown mouse models, Wang *et al.* demonstrated the involvement of mTORC2 in the regulation of ECs proliferation and angiogenesis both *in vitro* and *in vivo*, whereas the loss of function of mTORC1 gave only marginal effect on the ECs activities [12]. Hoang *et al.* have further shown that PP242 is able to exert anti-MM effects on several MM cell lines as well as on primary PCs from three newly diagnosed MM patients [25].

The important role of the relationship between angiogenesis and PCs in MM pathogenesis and progression is well known [4, 47, 48, 52, 53]. The new anti-myeloma therapeutic approaches, i.e. bortezomib [5] and IMiDS, such as thalidomide [54] and lenalidomide [7], that target both BM PCs and angiogenesis, have been well established in MM.

In conclusion, mTORC2 is mainly involved in the pro-angiogenic properties of MM-ECs. The selective inactivation of mTORC2 on MM-ECs through RICTOR silencing, and dual mTOR inhibition by PP242 restrains angiogenesis both *in vitro* and *in vivo*. Therefore, PP242 seems to be a promising agent with direct anti-MM activity and with potential synergistic property with bortezomib and lenalidomide, two well established anti-myeloma and anti-angiogenic drugs.

## MATERIALS AND METHODS

### Patients

Patients fulfilling the International Myeloma Working Group (IMWG2003 [55]) diagnostic criteria for newly diagnosed MM (*n* = 47) and MGUS (*n* = 20) were studied. The study was approved by the Ethics Committee of the University of Bari “Aldo Moro” Medical School, and all patients provided their informed consent in accordance with the Declaration of Helsinki.

### Reagents

PP242 and rapamycin were purchased from Sigma-Aldrich (St Louis, MO), bortezomib from Janssen-Cilag (Cologno Monzese, Italy), and lenalidomide from Celgene Corporation (Milan, Italy). Dulbecco’s modified Eagle’s medium (DMEM), RPMI 1640, antibiotic/antimycotic, tripsyn/EDTA and phosphate-buffered saline (PBS) without Ca^2+^ and Mg^2+^, heat-inactivated fetal bovine serum (FBS) were obtained from Sigma-Aldrich.

### ECs isolation and characterization

Bone marrow aspirates from patients with MGUS and MM were centrifuged on Ficoll- Hypaque (Pharmacia Biotech, Uppsala, Sweden) gradient, and the separated mononuclear cells were left to adhere in complete medium (RPMI-1640 medium supplemented with 10% FBS). To isolate ECs, BM adherent stromal cells were harvested and incubated with magnetic microbeads coated with anti-CD31 antibody (Miltenyi Biotec, Bergisch Gladbach, Germany) according to manufacturer’s instructions. Factor VIII-related antigen, CD31, Vascular Endothelial Growth Factor Receptor 2 (VEGFR2), and Tie2 expression of freshly isolated MGUS-ECs and MM-ECs was verified by flow cytometry FACS Canto II (Becton Dickinson-BD, San Jose, CA, USA).

### Western blotting

Total protein lysate (35 μg) from MGUS-ECs and MM-ECs were separated on 4-12% NuPAGE^®^ gels (Invitrogen Corp.), electro-transferred to a polyvinylidene difluoride membrane (PerkinElmer Life Science Inc., Boston, MA, USA), and immunoblotted with anti-mTOR, anti-p-mTOR (Ser2448), anti-AKT, anti-p-AKT (Ser473), anti-pS6K1, anti-p-pS6K1 (Thr389), anti-RAPTOR, anti-ERK1/2 (p44/42), anti-p-ERK1/2 (Thr202/Thr204) (Cell Signaling Techology Inc., Danvers, MA, USA), anti-RICTOR (Abcam, Cambridge, UK), and anti-β-actin antibodies (Sigma-Aldrich). Then, the membranes were incubated with horseradish peroxidase-labeled secondary antibodies (Bio-Rad, Hercules, CA, USA). Immuno-reactive bands were visualized by enhanced chemiluminescence (LiteAblot extend substrate, Euroclone, Pero, Milan, Italy) and the Gel Logic 1,500 Imaging System (Eastman Kodak Co., Rochester, NY, USA), quantified with the Kodak Molecular Imaging Software. The expression bands were quantified as arbitrary optical density (OD) units.

### Real time RT- PCR

Total RNA was isolated using the RNeasy Micro kit (Qiagen, Venlo, Netherlands) and reverse transcripted into total cDNA with the iScript cDNA Synthesis Kit (Bio-Rad). Real time RT-PCR reactions were carried out with the “StepOne Real-Time RT-PCR System” (Applied Biosystems, Waltham, MA, USA), performed with specific primers for mTOR, RAPTOR and RICTOR using Taqman^®^ RT-PCR technology (Applied Biosystems). The gene expression (fold change) was measured with the comparative threshold cycle (Ct) method using glyceraldehyde-3-phosphate dehydrogenase (GAPDH) as endogenous control and the 2^-ΔΔCt^ formula [56].

### RICTOR siRNA transient transfection

MM-ECs (5x10^6^) were transiently transfected for 48h and 72h with 25 nM and 50 nM of siRNAs specific for RICTOR (target sequences: a) GGGAAUACAACUCCAAAUA; b) GCGAGCUGAUGUAGAAUUA; c) GAAGAUUUAUUGAGUCCUA; d) GACACAAGCACUUCGAUUA) and negative control scramble siRNAs (SMART-pool; Dharmacon RNA Technologies, Lafayette, CO, USA), or with the transfection reagent alone (Lipofectamine, RNAiMAX siRNA transfection reagent, Invitrogen Corp).

### Immunofluorescence

MM-ECs (5x10^3^) were cultured on chamber slides (Lab Tek II Chamber Slides, Thermo Scientific Fisher Scientific Inc, Waltham, MA, USA). To demonstrate the co-localization of mTOR with RAPTOR and RICTOR, cells were fixed with 2% paraformaldehyde, permeabilized with Triton-X100 and then incubated with p-mTOR (Ser2448 – immunohistochemistry, Cell Signaling Techology Inc., Danvers, MA, USA), RAPTOR (Merck Millipore, Darmstadt, Germany) and RICTOR (Abcam, Cambridge, UK). Then, cells were incubated with secondary antibodies conjugated with fluorescein isothiocyanate (FITC) and tetramethylrhodamine (TRITC). For F-actin staining, MM-ECs treated with siRNA and PP242, were fixed with 2% paraformaldehyde, permeabilized with Triton-X100 and then incubated with phalloidin-FITC (Sigma-Aldrich). Nuclei were counterstained with 4’,6-diamidino-2-phenylindole (DAPI) at a concentration of 150 nM for 10 minutes (Invitrogen Corp).

### Wound healing assay

MM-ECs (4x10^4^) were grown to confluence on 24-well plates in complete medium. A wound was made by scraping the cell monolayer with a sterile pipette tip. Medium was removed and the cells were cleaned with PBS. Then, they were exposed to serum-free medium (SFM) as negative control, complete DMEM (as positive control) and PP242 at 10, 50, 100 and 200 nM. In silencing experiments, the scratch was made at 56h. After 16h, cells were fixed and stained. Cell migration was determined by counting the MM-ECs migrated into the “wound”, and cells are indicated as the percentage of relative wound closure compared with control.

### Chemotaxis and chemo-invasion assays

To evaluate the chemotaxis ability of MM-ECs, they were tested (5x10^4^) in a Boyden micro-chamber assay toward DMEM alone (negative control), DMEM with 1.5% FBS added with VEGF and FGF-2 (both at 10 ng/ml; Miltenyi Biotec, Bergisch Gladbach, Germany) as chemo-attractants (positive control) in RICTOR silencing experiments and, in DMEM with 100 nM of PP242 in experiments with the mTOR inhibitor. In the same conditions, the chemoinvasive ability of MM-ECs was evaluated using BD BioCoat™ Matrigel™ Invasion Chamber (BD Biosciences, Milan, Italy). After 16h at 37°C, the migrated/invaded cells were fixed, stained and counted at 400X on the EVOS inverted microscope (EuroClone, Pero, Milan, Italy).

### Adhesion assay

MM-ECs were treated with 25 nM siRICTOR for 72h or with 100 nM PP242 for 48h, stained with Calcein AM for 1h, and then plated (1x10^3^ cells per well) in quadruplicate in 96-well fibronectin-coated (10 μg/mL) plate. After 30 minutes, non-adherent cells were washed away and the rate of adherent cells was evaluated reading fluorescence at 495 nm by VICTOR™ X3 Multilabel Plate Reader (PerkinElmer Inc., Waltham, MA, USA).

### Capillarogenesis assay on matrigel^®^

MM-ECs pre-transfected with siRICTOR or pre-treated with drugs (rapamycin, PP242, lenalidomide, bortezomib) were plated (3.5x10^4^) on 48-well plates coated with Matrigel^®^ (BD Biosciences) in SFM. After 16h, the skeletonization of the mesh was followed by measurement of mesh areas and vessel length in three randomly chosen fields with the EVOS microscope at 10X.

### Apoptosis and proliferation assays

The apoptotic effect of PP242 was analysed by using the Annexin-V-PE/7aminoactinomycin-D assay (BD) according to manufacturer’s instructions. Samples were evaluated by flow cytometry by the FACS Canto II. To determine the effect of PP242 on MM-ECs proliferation, cells were labeled with carboxyfluorescein succinimidyl ester (CFSE) by using CellTrace cell proliferation kit (Molecular Probes Inc.) according to manufacturer’s instructions. Labeled MM-ECs (4x10^4^) were treated with PP242 at different concentration (10÷200 nM) for 72h, then evaluated by the FACS Canto II.

### Zymography

CM from 5x10^5^ MM-ECs, treated or not with 100 nM PP242 for 48h, were collected, centrifuged at 450 g at 4°C for 5 minutes to eliminate cell debris, and concentrated to 1x10^6^ cells/ml at 950 g at 4°C for 60 minutes. The CM were mixed with sodium dodecyl sulphate buffer under non-reducing conditions, and run on Novex^®^ Zymogram gelatine gel (Invitrogen Corp.) at 125V for 90 minutes. After electrophoresis, the enzyme was renatured by incubating the gel in Zymogram renaturing buffer containing a non-ionic detergent. The gel was equilibrated in Zymogram developing buffer, and then stained and destained following manufacturer’s instructions (Invitrogen Corp.). Regions of protease activity appeared as clear bands against a dark blue background.

### Cytokine measurement

CM by seeding 2x10^5^ cells treated or not with PP242 (100 nM) were obtained as reported above. Cytokines were measured by using Q-Plex™ Array Human Angiogenesis Antigen (Quansys Biosciences, Logan, UT, USA) that detects ANG-2, FGF-2, HGF, IL-8, PDGF-BB, TIMP-1 and -2, TNF-α and VEGF, according to the manufacturer’s instructions. Secreted levels of cytokines were quantified through Q-View Software (Quansys Biosciences).

### CAM assay

Fertilized white Leghorn chicken eggs were incubated at 37°C and constant humidity. On day 3, the shell was opened and 2-to-3 mL of albumen removed to detach the CAM. On day 8, the CAMs were implanted with 1 mm^3^ sterilized gelatin sponges (Gelfoam Upjohn Co., New York, NY, USA), loaded with SFM alone (negative control), or with conditioned medium (CM) of treated/untreated MM-ECs for 24h and 48h. On day 12, the angiogenic response was evaluated as the number of vessels converging toward the sponge at 50X and photographed *in ovo* by a stereomicroscope (Olympus Italia Srl, Segrate, Milan, Italy).

### Matrigel^®^ plug assay

The anti-angiogenic activity of mTOR inhibitor PP242 was evaluated *in vivo* in the murine Matrigel^®^ plug assay. Seven-week-old NOD/SCID female mice (Envigo, Bresso, Italy) were injected subcutaneously, into the flank, with 300 μL of liquid Matrigel (Cultrex^®^ BME Growth Factor Reduced, Trevigen, Helgerman CT, Gaithersburg) containing 500 ng of FGF-2 and 500 ng of VEGF (Miltenyi Biotec, Bergisch Gladbach, Germany) as positive control, PBS added with CM of MM-ECs treated and not treated with PP242, and PBS alone as negative control. To evaluate PP242 direct effect on the mTOR inhibition, a group of mice injected with pro-angiogenic cytokines were treated or not intraperitoneally (IP) with PP242 50mg/Kg twice a week [57]. Ten days after injection, mice were sacrificed. Harvested plugs were embedded in Tissue Tec Optimal Cutting Temperature (Sakura Finetek USA, Torrance, CA, USA) and analyzed by immunofluorescence microscopy for mouse CD31 expression (Abcam, Cambridge, UK). Animal experiments were approved by local animal ethics committee (OPBA) at the University of Bari “Aldo Moro”, and were executed in accordance with national guidelines and regulations.

### Statistical analysis

This was performed using GraphPadPrism5 software. Results were analyzed using the Wilcoxon signed-rank test. P < 0.03 was considered statistically significant.

## SUPPLEMENTARY MATERIALS FIGURES


