# Supplementary material for: Inhibition of mTOR complex 2 restrains tumor angiogenesis in multiple myeloma
Source: Oncotarget. 2018 Apr 17;9(29):20563–77. doi: 10.18632/oncotarget.25003 (PMC5945497; doi:10.18632/oncotarget.25003)
Supplement: Supplementary file 1 [file oncotarget-09-20563-s001.pdf]

Inhibition of mTOR complex 2 restrains tumor angiogenesis in multiple myeloma

SUPPLEMENTARY MATERIALS

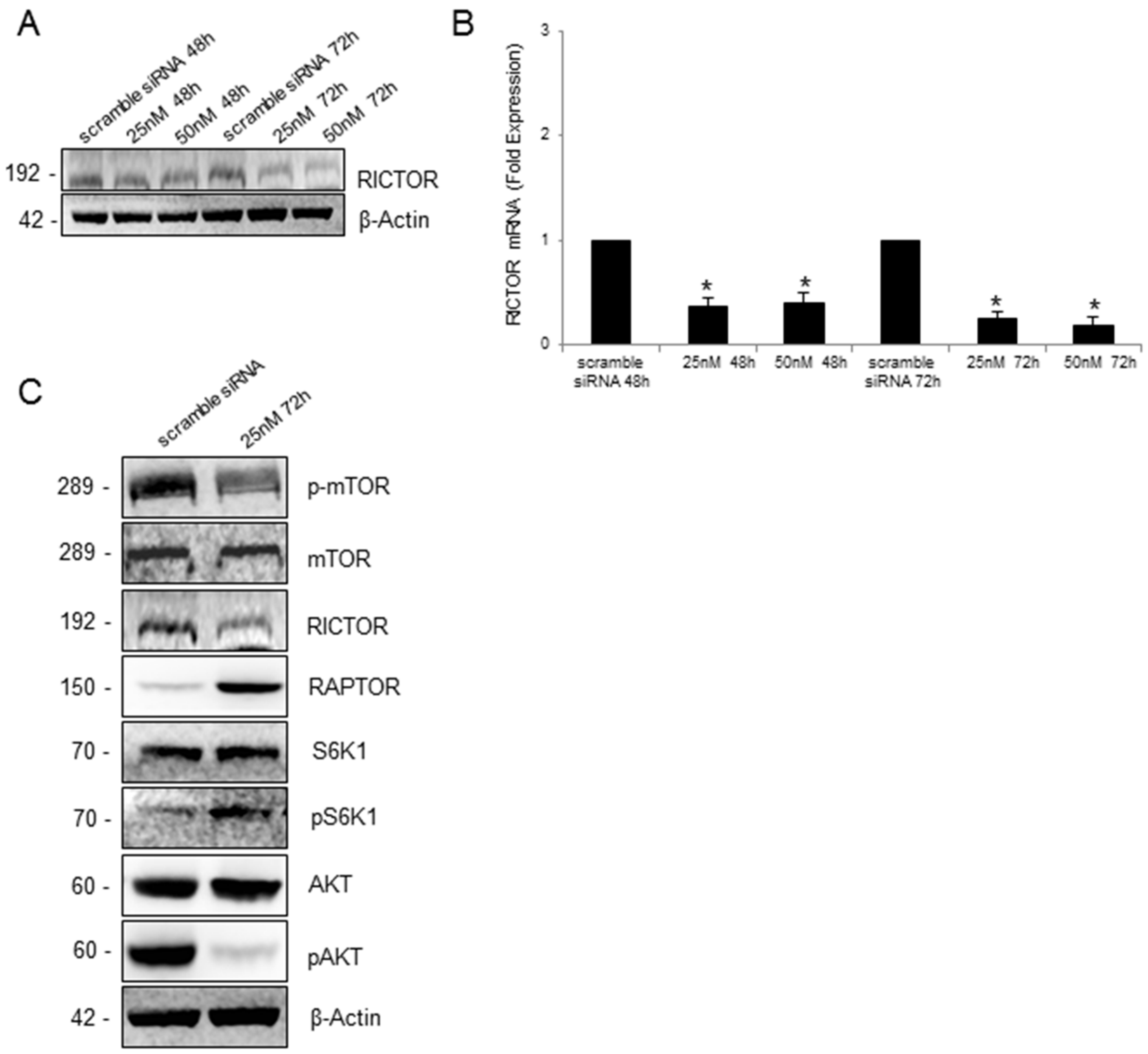

Supplementary Figure 1: Time- and dose-finding for RICTOR knock-down. **(A)** Total proteins from MM-ECs (n=8) treated with scramble siRNA or with RICTOR siRNA 25 nM or 50 nM for 48h and 72h were analyzed by Western blotting for RICTOR expression. Representative images from 8 independent experiments are shown. **(B)** Total mRNA was extracted from MM-ECs (n=8) treated with scramble siRNA or with RICTOR siRNA (25 nM or 50 nM for 48h or 72h). RICTOR expression was analyzed by Real-Time RT-PCR. Data are expressed as mean±SD. **(C)** Total protein from MM-ECs (n=8) treated with scramble siRNA or with RICTOR siRNA (25 nM for 72h) were analyzed by Western blotting for mTOR, p-mTOR, RICTOR, RAPTOR, S6K1, p-S6K1, AKT and p-AKT expression. Representative image of a single MM-ECs sample. *p < 0.03 by Wilcoxon signed-rank test.

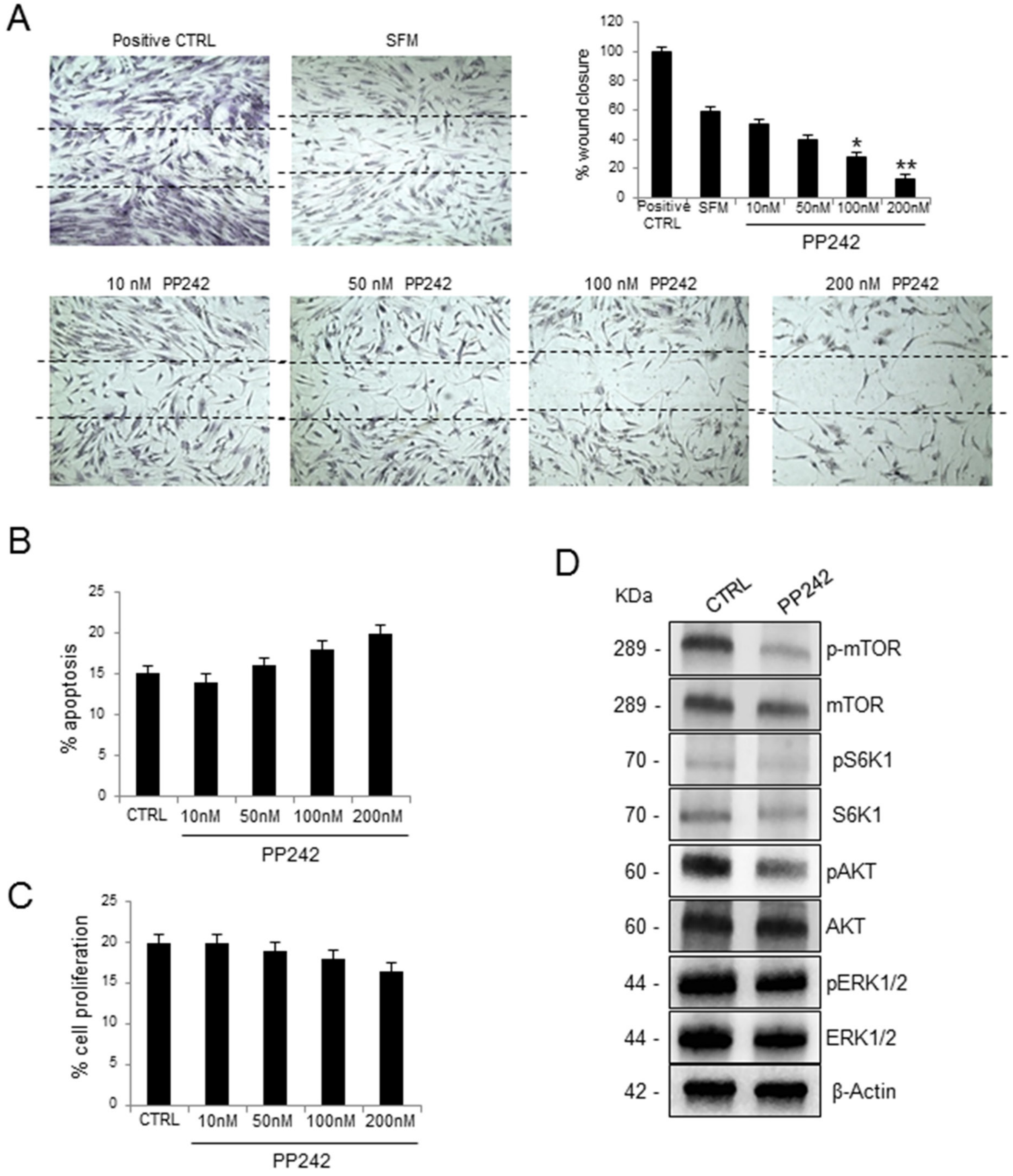

Supplementary Figure 2: Dose-finding for dual mTOR inhibitor PP242. **(A)** ConfluentMM-ECs (n=6) seeded in 24-well plates were treated with increasing concentrations of PP242 (10÷200 nM), scratched and tested in wound-healing assay in order to assess the optimal concentration of PP242 able to inhibit MM-ECs spontaneous migration. Original magnification 200X. Representative images from 6 independent experiments are shown. Bar graph represents the relative closure of the wound and data are expressed as mean±SD. The positive control was established as 100%. **(B-C)** MM-ECs (n=6) were treated with increasing concentrations of PP242 (10÷200 nM) for 72h and analyzed for (B) cell apoptosis usingAnnexin V/7AAD assay and (C) cell proliferation usingCFSE staining. Data are expressed as mean±SD. **(D)** Total protein from untreated MM-ECs as control (CTRL) or treated with PP242 (100 nM for 48h) were analyzed by Western blotting for mTOR, p-mTOR, S6K1, p-S6K1, AKT, p-AKT, ERK1/2 and p-ERK1/2 expression. Representative images from 6 independent experiments are shown. $^{*}p < 0.03$ and $^{**}p < 0.003$ by Wilcoxon signed-rank test.
